# Frequency and significance of IgG4 immunohistochemical staining in liver explants from patients with primary sclerosing cholangitis

**DOI:** 10.1111/iep.12076

**Published:** 2014-04-18

**Authors:** Sandra Fischer, Palak J Trivedi, Stephen Ward, Paul D Greig, George Therapondos, Gideon M Hirschfield

**Affiliations:** * Department of Liver Pathology, University of TorontoToronto, ON, Canada; † National Institute for Health Research (NIHR) Birmingham Liver Biomedical Research Unit (BRU), University of BirminghamBirmingham, UK; ‡ Department of Surgery, University of TorontoToronto, ON, Canada; § Multi-Organ Transplant Institute, Ochsner Medical CenterNew Orleans, LA, USA

**Keywords:** bilary disease, dominant stricture, liver transplantation, pancreatitis

## Abstract

Dense tissue infiltrates of IgG4^+^ plasma cells >50/high-powered field (HPF) are purportedly highly specific for IgG4-related disease. However, the frequency and significance of liver-infiltrating IgG4^+^ plasma cells in primary sclerosing cholangitis (PSC) applying these cut-offs has not been determined. We sought to determine the incidence of intrahepatic IgG4-positive staining in PSC patients undergoing transplantation, correlating findings with clinical parameters. Immunohistochemical staining was performed on liver explants obtained between 1991 and 2009. Of 122 explants obtained, hilar IgG4^+^ staining was found to be mild (10–29 IgG4^+^ cells/HPF) in 23.0%, moderate (30–50/HPF) in 9.0% and marked (>50/HPF) in 15.6%. Marked hilar lymphoplasmacytic infiltration was significantly associated with marked hilar IgG4^+^ staining (*P* < 0.001). No patient had marked peripheral IgG4^+^ staining, although mild and moderate staining was observed in 24.5% and 3.3% respectively. Marked hilar IgG4^+^ staining was significantly associated with the presence of dominant biliary strictures (*P* = 0.01) and need for biliary stenting (*P* = 0.001). There did not, however, exist any significant differences in the age at PSC diagnosis, presence of inflammatory bowel disease or extrahepatic autoimmune disease, frequency of cholangiocarcinoma, interval between diagnosis and transplantation, or post-transplant PSC recurrence or survival. Of 51 control liver sections (PBC = 18; HCV = 19; HBV = 8; AIH = 6), none had marked or moderate hilar IgG4^+^ staining, whereas mild staining was seen in only 10% (*P* < 0.001). Marked (>50/HPF) hilar IgG4^+^ lymphoplasmacytic infiltration is frequently observed in PSC and associated with the presence of dominant biliary strictures. However, unlike serum IgG4^+^_,_ this does not seemingly associate with clinical disease course.

Primary sclerosing cholangitis (PSC) is a chronic progressive disease of unknown aetiology characterized by fibrosclerotic destruction of the bile ducts resulting in multifocal stricturing of the biliary tree (Mendes & Lindor 2010; Hirschfield *et al*. 2013). Orthoptic liver transplantation (OLT) currently remains the only curative option; however, disease recurrence occurs in approximately 30% (Farges *et al*. 1995; Solano *et al*. 2003; Maheshwari *et al*. 2004).

Immunoglobulin G4 (IgG4)-associated cholangitis (IAC) often has similar cholangiographic features to PSC (Abdalian & Heathcote 2006; Deheragoda *et al*. 2007; Umemura *et al*. 2007; Esposito *et al*. 2008; Webster *et al*. 2009; Culver & Bateman 2012) and sits within a broader family of IgG4-related systemic disease of which autoimmune pancreatitis (AIP) is the commonest manifestation (Stone *et al*. 2012). Despite sharing many clinicopathological features with PSC, an important distinction to recognize is that IAC is responsive to treatment with corticosteroids (Björnsson 2008) and does not share such a strong association with inflammatory bowel disease (IBD) (Ohara *et al*. 2012), although it has recently been reported that intestinal mucosal IgG4^+^ plasma cells are particularly increased in patients with colitis in the context of PSC (Raina *et al*. 2013). An international consensus group recently published their diagnostic criteria for IAC incorporating similar histological, radiological, serological and clinical findings to those found in the Mayo Clinic's HISORt criteria for AIP (Chari 2007; Ohara *et al*. 2012). Two key diagnostic features of IAC are an elevated serum IgG4 level and the presence of tissue-infiltrating IgG4-positive plasma cells on biopsy. However, ∼10% of patients with PSC are reported to have elevated serum IgG4 levels and thought to represent a subpopulation with higher Mayo risk score, higher serum ALP values, a greater propensity to develop liver cirrhosis (Mendes *et al*. 2006) and reduced colectomy-free survival (Navaneethan *et al*. 2013). Tissue-infiltrating IgG4-positive plasma cell deposition has also been reported in PSC liver, and in some studies, this has been proposed to signify a more aggressive clinical course with shorter time to transplantation and a higher likelihood of disease recurrence (Zhang *et al*. 2010). However, the appropriate ‘cut-off’ point used in differentiating true IgG4^+^_-_related cholangitis/pancreatitis from other pro-inflammatory conditions such as PSC is unclear. According to the ‘Consensus Statement on the Pathology of IgG4 disease,’ if a liver biopsy contains >10 IgG4-positive plasma cells per high-powered field (HPF) and at least one histological feature (dense lymphoplasmacytic infiltrate/storiform fibrosis/obliterative phlebitis), this finding would be probable of IgG4-related disease (Deshpande *et al*. 2012), whereas the finding of >50 IgG4^+^ plasma cells/HPF (Boston criteria) has been reported to have the highest specificity (Deshpande *et al*. 2012). Thus far, no study has assessed the significance of finding liver-infiltrating IgG4^+^ plasma cells in PSC using the latter cut-off.

The primary aims of our study were to validate the prevalence of IgG4-positive plasmacytic tissue infiltration in patients undergoing liver transplantation for PSC using the revised Boston criteria and evaluate for any inherent phenotypic differences *vs*. those who stained IgG4-negative.

## Materials and methods

A case series review was performed on all patients with PSC undergoing liver transplantation at University Health Network, Toronto, between 1991 and 2009. Explanted specimens from unrelated aetiologies leading to chronic liver disease were identified from our pathology database and served as a comparator group. Institutional ethics approval was obtained.

### Clinical data

Clinical records were reviewed to establish the age at PSC presentation, ductal phenotype (intrahepatic *vs*. combined intra- and extrahepatic disease), presence of dominant strictures – defined as stenosis of the common bile duct (CBD) with a diameter ≤1.5 mm, or of the left or right hepatic duct with a diameter ≤1 mm; time from PSC diagnosis to transplantation, presence/phenotype of IBD, history of pancreatitis, evidence suggestive of extrahepatic IgG4-related diseases or concomitant extra-hepatopancreatobiliary autoimmune conditions, and/or the presence of clinically unsuspected, incidental cholangiocarcinoma in the explant. Patient survival and evidence of recurrent PSC post-transplant were also evaluated. Recurrent PSC was suspected based on abnormal liver biochemistry post-transplant and confirmed with diagnostic cholangiography. Allograft biopsy was performed in cases of diagnostic uncertainty.

All patients undergoing transplantation were subject to full clinical, laboratory and radiological assessment prior to the procedure including a detailed clinical examination, laboratory work-up, cholangiogram and cross-sectional imaging. On review, any individual with radiological and/or clinical evidence suggestive of systemic extra-hepatopancreatobiliary (HPB) manifestations of IgG4-related disease according to HISORt criteria (Chari 2007) was excluded from the study.

### Histopathology

**Figure 1:**
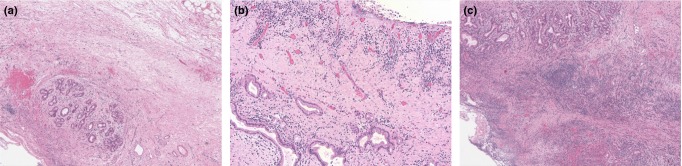
Classification of severity of hilar lymphoplasmacytic inflammation. Sections of liver explants (hilum) with PSC showing variable lymphoplasmacytic inflammation: (a) mild, (b) moderate, and (c) severe. (HE stain, ×100 magnification).

Formalin-fixed, paraffin-embedded tissues sampling the liver hilum were retrieved, and haematoxylin/eosin and Masson's trichrome staining performed on all sections. Given that percutaneous liver biopsies routinely sample the peripheral liver parenchyma, paired peripheral sections were also obtained for analysis. All cases were reviewed by a histopathologist with expertise in hepatobiliary disease blinded entirely to clinical characteristics and patient outcome. Hilar lymphoplasmacytic infiltration was characterized into three strata: mild, moderate and severe (Figure1). Briefly, mild infiltration was defined by scattered inflammatory aggregates with few lymphocytes and plasma cells; moderate infiltration was considered as such if dense focal lymphoplasmacytic aggregates were identified in the hilum or around bile ducts; severe infiltration was defined by the presence of a diffuse and dense hilar collection of lymphocytes and plasma cells.

### Immunohistochemistry

Specimens were immunohistochemically stained with anti-IgG4 antibody (mouse monoclonal, Cat. No.: 05-30800, clone HP6025; Life Technologies Inc., Burlington, ON, Canada) at a dilution of 1:2000 (v/v) and one hour incubation. Briefly, 4-μm sections were dewaxed in five changes of xylene and brought down to water through graded alcohols. Pretreatment with 1% pepsin (P7125; Sigma-Aldrich Canada Co., Oakville, ON, Canada) in 0.01 NaHCl (pH 2.0) was performed for 15 min (37 °C), and endogenous peroxidase blocked with 3% H_2_O_2_. The detection system used was Biogenex Super-Sensitive Polymer Kit (QD410-YAX) with colour development performed using freshly prepared NovaRed (Cat. No. SK-4800; Vector Laboratories Inc., Burlington, ON, Canada). Finally, sections were counterstained lightly with filtered Mayer's haematoxylin, dehydrated in alcohols, cleared in xylene and mounted with Permount medium (Cat. No.: SP15-500; Fisher Scientific Company, Ottawa, ON, Canada).

**Figure 2:**
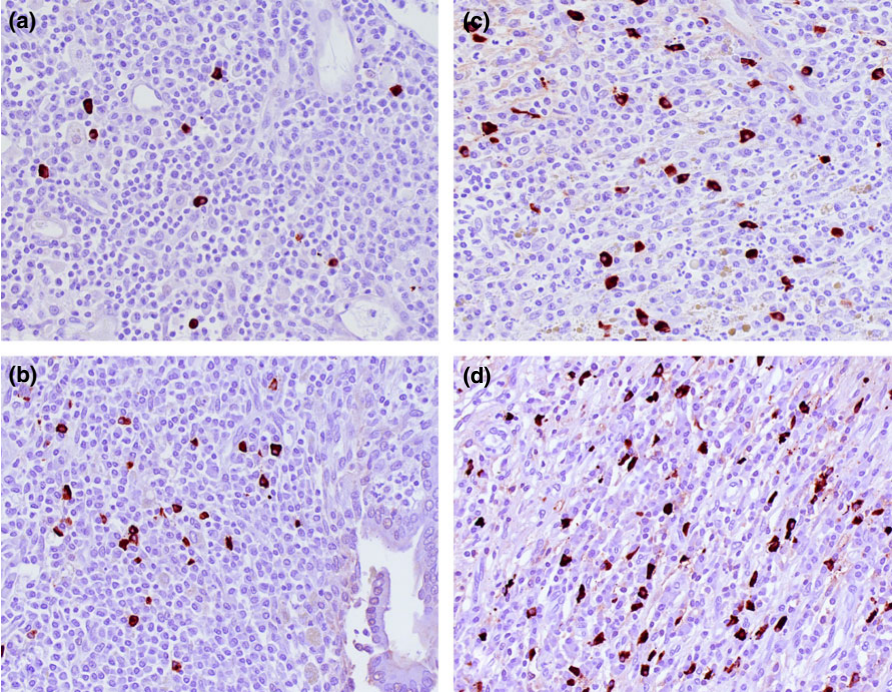
Stratification of IgG4^+^ immunostaining. IgG4 immunostaining of liver explants (hilum) with PSC showing: (a) focal (negative) immunoreactivity, (b) mild immunoreactivity (c) moderate immunoreactivity, and (d) marked immunoreactivity (×400 magnification).

To quantify the degree of IgG4 positivity within any given tissue section, the field containing the highest number of IgG4^+^ cells was counted and polychotomized into 4 strata: negative (<10 IgG4^+^ plasma cells/HPF), mild (10–29 cells/HPF), moderate (30–50 cells/HPF) and marked (>50 cells/HPF; Figure2).

### Statistical analysis

IgG4-positivity was correlated with the aforementioned clinical parameters and degree of hilar lymphoplasmacytic inflammation. Mann–Whitney-U nonparametric method was used to determine whether significant differences existed between groups. Differences in nominal data were compared by chi-squared test or Fisher's exact test when the number was less than 5 in any given cell of a 2 × 2 table. The level of significance was set at *P* < 0.05. Statistical analysis was performed using spssv21.

## Results

### Patient characteristics

Over an 18-year period, 157 patients with PSC underwent liver transplantation, of which explant tissue was readily available in 122 cases (89 male). The median age of PSC diagnosis was 36 years (range 10–67) with a median interval from diagnosis to transplant of 6 years (8 months–24 years). 27.9% (*n* = 34) had only intrahepatic involvement, whereas 67.2% of patients (*n* = 82) had coexisting IBD, and 20.5% (*n* = 25) a history of extra-hepatopancreatobiliary (HPB) autoimmune disease. Of all patients transplanted, 5 (4.1%) were diagnosed as having cholangiocarcinoma *in-situ* (explant) and 13 (10.7%) developed evidence of recurrent PSC.

The comparator group (*n* = 51) comprised 19 patients transplanted for chronic viral hepatitis type C (HCV) infection, eight with chronic viral hepatitis B (HBV) infection, 18 with primary biliary cirrhosis (PBC; *n* = 18), and six transplanted for autoimmune hepatitis (AIH; *n* = 6).

### The severity of hilar IgG4-positive immunostaining is associated with the severity of hilar lymphoplasmacytic infiltration in PSC

End-stage chronic biliary disease with dense periductal concentric fibrosis and ductopenia was confirmed in all PSC cases undergoing transplantation. Of the 122 PSC explants studied, 47.5% (*n* = 58) had positive IgG4 immunohistochemical staining in the hilar tissue, of which 23.0% (*n* = 28) had mild staining, 9.0% (*n* = 11) had moderate staining and 15.6% (*n* = 19) had marked staining. Hilar lymphoplasmacytic infiltration was marked in 52.9% (*n* = 64), moderate in 28.1% (*n* = 34) and mild in 19.0% (*n* = 23) of PSC patients. Neither storiform fibrosis nor significant obliterative phlebitis [which are also characteristic features of IAC (Ohara *et al*. 2012)] was identified in our PSC cohort.

**Figure 3:**
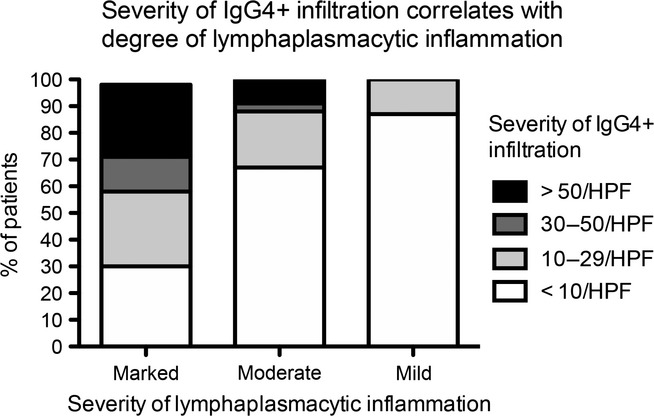
IgG4^+^ staining and degree of hilar lymphoplasmacytic inflammation in explanted PSC liver tissue. Patients with positive IgG4 immunohistochemical staining were significantly more likely to have marked lymphoplasmacytic infiltration (*P* < 0.001).

Patients with mild or moderate hilar lymphoplasmacytic infiltration did not have statistically more positive staining for IgG4 (*P =* 0.12), unlike patients with marked hilar lymphoplasmacytic infiltration, who were significantly more likely to have positive immunohistochemical IgG4 staining of any degree compared to those with lesser degrees of hilar lymphoplasmacytic infiltration (OR: 15.6; 4.2–58.0, *P* < 0.001). This retained significance when restricting the analysis to those with >50 IgG4^+^ plasma cells/HPF (OR 6.0; 1.6–21.9, *P* < 0.001, Figure3).

Only five non-PSC cases (3 HCV, 1 HBV, 1 PBC) had positive hilar IgG4 immunohistochemical staining, and this was of a mild degree only; no patient in the control group was identified as having marked or moderate staining. The frequency of hilar IgG4^+^ staining was significantly lower compared to that found in PSC explants (*P* < 0.001).

### The severity and pattern of hilar IgG4^+^ staining does not correlate with that observed in peripheral liver parenchyma in PSC

Thirty-four PSC patients (27.9%) also had positive IgG4 immunohistochemical staining in the peripheral liver parenchyma; 24.6% (*n* = 30) had mild staining and 3.3% (*n* = 4) moderate staining. There was no significant association between severities of peripheral IgG4 staining and hilar IgG4 staining, neither did there exist an association between the severity of hilar lymphoplasmacytic infiltration and the severity of peripheral IgG4^+^ staining (data not shown). Four patients (7.8%) in the comparator group (3 HCV, 1 PBC) had evidence of peripheral IgG4^+^ staining (*P* < 0.001) although this was of a mild degree only.

### Patients with PSC and positive hilar IgG4^+^ staining are more commonly men and have a previous history of acute pancreatitis

In our PSC cohort, the presence of any hilar IgG4-positive immunostaining (>10 1gG4^+^ plasma cells/HPF) was more common in men (83.1% *vs*. 65.1% in the IgG4-negative group; *P =* 0.04) and associated with a previous history of acute pancreatitis (13% *vs*. 1.7%; *P =* 0.03). On retrospective evaluation, no radiological features indicative of AIP were identified in this subgroup. On comparing with patients who stained negative for IgG4, no statistically significant differences existed with respect to age at PSC diagnosis, presence/phenotype of coexisting IBD or extrahepatic autoimmune disease, ductal phenotype, frequency of cholangiocarcinoma, age at time of transplantation, interval between PSC diagnosis and transplantation (Figure S1), post-transplant PSC recurrence or post-transplant survival (Table 1).

**Table 1:** Hilar IgG4 immunohistochemical staining and clinical characteristics

	IgG4^+^ No. cases (%)*	IgG4^−^ No. cases (%)	*P-*value
Total	59 (48.4)	63 (51.6)	
Male gender	49 (83.1)	41 (61.5)	0.04
History of acute pancreatitis	7 (87.5)	1 (12.5)	0.03
Concomitant autoimmune disease†	13 (24.1)	12 (19.0)	0.07
History of IBD	40 (67.8)	42 (65.6)	0.95
Median age (years) at PSC diagnosis	36; range 16–67	39; range 13–63	0.23
Intrahepatic ductal involvement only	18 (31.0)	16 (25.0)	0.46
Dominant stricture	18 (31.0)	17 (26.6)	0.59
Previous biliary stenting	20 (34.5)	13 (20.3)	0.08
Pretransplant MELD score	14.6; range 9.2–26.7)	14.3; range 8.2–25.1	0.48
Cholangiocarcinoma‡	2 (3.8)	3 (5.5)	1.00
Median age (years) at transplant	40; range 18–61	44; range 21–66	0.15
Median time to transplant (years) after PSC diagnosis	6; range 0–24	6; range 0–22	0.40
PSC recurrence after ransplant	8 (13.6)	5 (7.8)	0.46
All cause mortality after transplant	13 (22.0)	14 (21.9)	0.84

* **>**10 IgG4^+^ plasma cells/high-powered field.

† Excluding inflammatory bowel disease (IBD).

‡ Diagnosed on explant.

### Individuals with PSC and marked (>50/HPF) hilar IgG4^+^ staining more frequently have a dominant stricture

When restricting comparative analysis using the cut-offs provided by the recent Boston criteria for IgG4-associated systemic disease, no significant gender differences were observed between those with marked hilar IgG4^+^ staining *vs*. those with <50 IgG4^+^ plasma cells/HPF (*P* = 0.25), and despite a trend remaining with respect to previous episodes of acute pancreatitis (*P* = 0.08), this failed to retain statistical significance. Perhaps most strikingly, patients with moderate or marked hilar IgG4^+^ infiltration were significantly more likely to have a dominant biliary stricture than those with lesser degrees of IgG4^+^ infiltration (43.3% and 52.6% *vs*. 24.3%; *P* = 0.04 and *P =* 0.01 respectively) and required biliary stenting more commonly (43.3% and 57.9% *vs*. 21.3%; *P* = 0.02 and *P* = 0.001 respectively).

However, there was no significant difference in the frequency of cholangiocarcinoma, median age at PSC diagnosis, median age at transplantation, pretransplant MELD score, interval to transplantation following PSC diagnosis, frequency/phenotype of coexisting IBD, incidence of post-transplant PSC recurrence and all cause mortality after transplantation between groups (Table 2).

**Table 2:** Patients with marked hilar IgG4^+^ staining do not have a different clinical phenotype to those with lesser degrees of staining

	>50 IgG4^+^ plasma cells/HPF No. cases (%)	<50 IgG4^+^ plasma cells/HPF No. cases (%)	*P-*value
Total	19 (15.6)	103 (84.4)	
Male gender	16 (84.2)	73 (70.9)	0.25
History of acute pancreatitis	3 (15.8)	5 (4.10)	0.08
Concomitant autoimmune disease*	2 (10.5)	23 (22.3)	0.05
History of IBD	16 (84.2)	65 (63.1)	0.08
Median age (years) at PSC diagnosis	37.5; range 11–68	37; range 10–63	0.93
Intrahepatic ductal involvement only	6 (31.6)	28 (27.2%)	0.71
Dominant stricture	10 (52.6)	25 (24.3)	0.01
Previous biliary stenting	11 (57.9)	22 (21.4)	0.001
Pretransplant MELD score	14.3; range 9.2–22.5	14.4; range 8.2–26.7	0.99
Cholangiocarcinoma‡	0 (0.0)	5 (4.9%)	0.32
Median age (years) at transplant	40.5; range 18–70	43; range 18–66	0.85
Median time (years) to transplant after PSC diagnosis	7; range 1–25	6; range 2–22	0.60
PSC recurrence after transplant	3 (15.6%)	8 (7.8%)	0.27
All cause mortality after transplant	3 (15.6%)	23 (22.3%)	0.84

a Excluding inflammatory bowel disease (IBD).

b Diagnosed on explant.

## Discussion

PSC is characterized histologically by chronic cholestasis, inflammation and obliterative biliary fibrosis eventually leading to cirrhosis. Presently, it is devoid of effective intervention other than transplantation, and efforts continue to rationalize pathophysiologic understanding with the goal of identifying themes for intervention that account for patient heterogeneity. The repeated observation of detecting elevated serum IgG4 levels in a subgroup of PSC patients has triggered interest in defining IgG4 contributions to disease presentation as well as clinical outcome. In IAC and other conditions falling under the ‘umbrella’ of IgG4-related systemic disease, there exist key histopathological features comprising recognized diagnostic criteria (Chari 2007; Deshpande *et al*. 2012; Ohara *et al*. 2012), which are distinct from changes observed in PSC. The morphological appearances of IgG4-related disease, though highly characteristic, require immunohistochemical confirmation with immunostaining of IgG4-bearing plasma cells, and although all immunoglobulin subclasses may be represented within involved tissue, IgG4 predominates the histological picture yielding an increased IgG4:total IgG ratio. The optimal value at which tissue-infiltrating IgG4^+^ staining differentiates IgG4-related HPB disease from other diagnoses such as PSC is heavily debated; however, the recent Boston criteria suggest that >50 IgG4^+^ plasma cells/HPF is highly specific for the former.

Herein, we illustrate that approximately half of all transplanted PSC patients have some evidence of hilar IgG4-positive staining in their explanted liver specimens. All patients had developed end-stage chronic biliary disease as evidenced by dense periductal concentric fibrosis, but without further evidence to support a formal diagnosis of IAC. The PSC group staining positive for hilar IgG4 plasma cells was more likely to be men and have a prior history of acute pancreatitis. This is noteworthy given that IAC is also frequently associated with AIP (Ohara *et al*. 2012), but also given our recently published study showing that PSC patients with an elevated serum IgG4 are also more often men with a previous episode of pancreatitis (Alswat *et al*. 2012). Our study also represents one of the first attempts to address the significance of IgG4^+^ plasma cells in PSC liver whilst applying the cut-offs deemed ‘highly specific’ used for diagnosing IAC as provided by the Boston criteria. In doing so, we were able to demonstrate that ∼16% of PSC patients undergoing transplantation have evidence of marked (>50/HPF) IgG4^+^ hilar infiltration, a finding associated with the severity of hilar lymphoplasmacytic inflammation. These results echo those published by Zhang *et al*. (2010). Such patients were also more likely to have developed dominant biliary strictures and a need for subsequent biliary intervention, itself a risk factor for developing acute pancreatitis. In diseases other than PSC, IgG4^+^ staining was, however, much less apparent.

Zen *et al*. (2007) recently reported finding IgG4-positive cellular staining in 29% of PSC liver explants and in keeping with our observations found no appreciable differences in the frequency of cholangiocarcinoma or coexisting autoimmune disease, time to transplant, post-operative mortality or disease recurrence rate post-transplant between IgG4-positive and IgG4-negative PSC patients. We did, however, observe a significant difference in the incidence of dominant biliary strictures which may reflect the application of using different cut-offs for defining IgG4-positive staining – ours being the first to assess the implications of >50 IgG4^+^ plasma cells/HPF in PSC. Whether there is a continuum of biliary disease with IAC and PSC belonging to the same spectrum remains unclear. The significance of finding IgG4^+^ tissue staining in PSC patients may perhaps represent the ‘end result’ of a local immune reaction around injured bile ducts in response to an as yet unidentified (auto)antigen, or to some intrinsic or extrinsic substrate present in bile, and offers a plausible explanation as to the observed association with dominant biliary strictures. Of interest, it is notable that a subset of patients with PSC and dominant biliary strictures may respond to immunosuppressive therapy (Erkelens *et al*. 1999) and warrant further characterization with respect to hilar IgG4^+^ status.

From a practical perspective, it is important to highlight the lack of association between peripheral and hilar samples. Although it is those with marked hilar lymphoplasmacytic inflammation who are most likely to stain positive for IgG4^+^ plasma cells, we have shown that there is poor correlation between hilar and peripheral IgG4-positive staining. This implies that peripherally targeted liver biopsies may not be particularly useful in terms of predicting whether a patient may have IgG4-associated cholangitis. Given the infrequency with which hilar biopsies are performed in routine practice, attempting selection of PSC patients for potential therapeutic trials of corticosteroid therapy, based on peripheral-tissue IgG4 positivity alone, should be discouraged.

The main limitations from our report stem from an absence of paired serum IgG4 readings. Given the retrospective nature of the study, the vast majority of included patients were transplanted in an era during which IgG4-related disease had yet to be recognized and whereby routine assessment of IgG4 was neither standard practice nor advocated by international guidelines. Nevertheless, we have recently reported on the frequency and clinical significance of elevated serum IgG4 elsewhere (Alswat *et al*. 2012). Moreover, Zhang *et al*. (2010), although using different cut-offs for determining high IgG4-positive staining, did reveal a positive correlation between serum IgG4 concentration and degree of IgG4-positive cellular infiltrate in the explanted livers of PSC patients, and there is merit in supporting a prospective approach to sample acquisition and evaluation in this regard. We were also unable to provide hilar IgG4/total IgG ratios; thus, it remains plausible that the observed IgG4 positivity reflects a non-specific effect of dense lymphoplasmacytic chronic inflammation. Furthermore, our cohort is clearly highly selected in those with advanced disease and is not therefore directly comparable to studies of patients with earlier disease that may or may not progress to transplantation.

In conclusion, hilar IgG4 staining is apparent on detailed immunohistochemical evaluation of liver grafts of patients with PSC in approximately 50% of cases. Such patients, particularly those with marked hilar IgG4^+^ staining, were more likely to have dominant strictures and need for biliary intervention. However, this in itself did not represent a subgroup with worse clinical outcome. It remains the case that understanding the drivers of IgG4 staining is still incomplete, and at present, the routine histological evaluation of peripheral IgG4 staining not clinically indicated.

## Supporting Information

**Figure S1**. The interval between PSC diagnosis and liver transplantation is not affected by the frequency of hilar IgG4^+^ lymphoplasmacytic infiltration.
