# Supplementary figures and images for: Frequency and significance of IgG4 immunohistochemical staining in liver explants from patients with primary sclerosing cholangitis
Source: Int J Exp Pathol. 2014 Apr 18;95(3):209–15. doi: 10.1111/iep.12076 (PMC4351857; doi:10.1111/iep.12076)

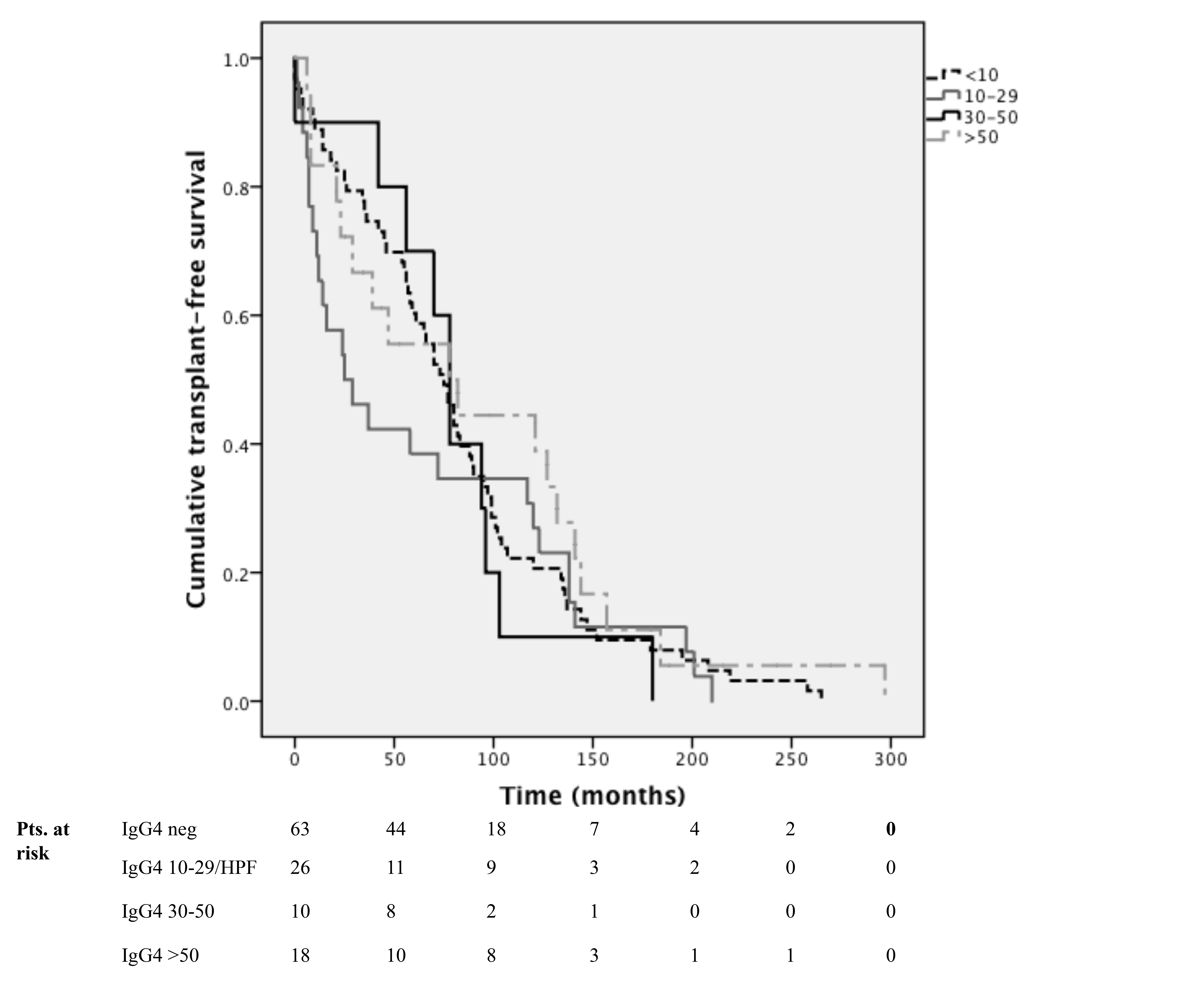

Supplement: Supplementary file 1 — Figure S1. The interval between PSC diagnosis and liver transplantation is not affected by the frequency of hilar IgG4+ lymphoplasmacytic infiltration. [file iep0095-0209-sd1.tif]
